# Screening of Circulation of Usutu and West Nile Viruses: A One Health Approach in Humans, Domestic Animals and Mosquitoes in Burkina Faso, West Africa

**DOI:** 10.3390/microorganisms10102016

**Published:** 2022-10-12

**Authors:** Bachirou Tinto, Didier Patinde Alexandre Kaboré, Thérèse Samdapawindé Kagoné, Orianne Constant, Jonathan Barthelemy, Alice Kiba-Koumaré, Philippe Van de Perre, Roch Kounbobr Dabiré, Thierry Baldet, Serafin Gutierrez, Patricia Gil, Dramane Kania, Yannick Simonin

**Affiliations:** 1 Pathogenesis and Control of Chronic and Emerging Infections, INSERM, University of Montpellier, 34394 Montpellier, France; 2 Centre MURAZ, Institut National de Santé Publique (INSP), Bobo-Dioulasso 01, Burkina Faso; 3 Institut de Recherche en Sciences de la Santé (IRSS), Bobo-Dioulasso 01, Burkina Faso; 4 Centre National de Transfusion Sanguine, Ouagadougou 01, Burkina Faso; 5 ASTRE Research Unit, CIRAD, INRAe, Montpellier University, 34398 Montpellier, France

**Keywords:** Usutu virus, West Nile virus, humans, domestic animals, mosquitoes, Burkina Faso

## Abstract

Usutu virus (USUV) and West Nile virus (WNV) are phylogenetically closely related arboviruses. These viruses mainly follow an enzootic cycle involving mosquitoes and birds, but they occasionally infect humans and other mammals, inducing neurotropic disorders. Since the discovery of USUV, only two human cases have been reported in Africa, including one in Burkina Faso in 2004. Since then, no studies have been conducted to measure the extent of the circulation of this virus in Burkina Faso, and no study regarding the circulation of WNV has been conducted. Our study aimed to determine the seroprevalence of USUV and WNV in blood donations and in animals (horses, dogs, chickens and pigeons) and to perform molecular screening in patients with febrile fever and in *Culex quinquefasciatus* and *Aedes aegypti* mosquitoes. The prevalence of USUV and WNV was studied by serological (ELISA and microneutralization tests) and molecular analyses (RT-qPCR) of mosquito, dog, domestic bird, horse, and human samples in Burkina Faso between 2019 and 2021. We detected a very active transmission of both viruses in Burkina Faso. WNV and USUV seroprevalence is particularly high in humans (19.16% and 14.17%, respectively) and horses (17.28% and 6.17%). Molecular screening did not detect WNV or USUV in the mosquito or human samples tested. Our study shows an active spread of USUV and WNV in Burkina Faso, especially for WNV. This study highlights the value of developing surveillance programs to better prevent, detect, and alert people to USUV and WNV circulation in both primary and incidental hosts.

## 1. Introduction

Usutu (USUV) and West Nile (WNV) viruses are arboviruses that are phylogenetically closely related, belonging to the Japanese encephalitis (JEV) serocomplex of the Flaviviridae family and the Flavivirus genus [1,2]. These viruses have a complex transmission cycle involving different species of birds as amplifying hosts, *Culex* mosquitoes as main vectors, and humans, horses, and other mammals as incidental hosts [3,4]. A transmission from human to human is rare and does not contribute to the circulation of these viruses, but several species of mosquitoes such as *Culex quinquefasciatus*, *Culex pipiens, Aedes albopictus*, and *Aedes japonicus* are involved in the transmission and circulation [5,6]. USUV was first isolated in 1959 from a *Culex neavei* mosquito collected near the Usutu river in Swaziland [7]. WNV was initially identified in 1937 in the West Nile Province of Uganda in a woman with a febrile syndrome [8]. These two viruses have long been considered viruses of African interest, given that they circulated originally in Africa, prior to their introduction to Europe and other continents. Major USUV epizootics affecting avifauna, associated with a large epidemic of WNV, were demonstrated in Europe in 2016 and in 2018 [2]. Although most USUV and WNV infections are asymptomatic, some patients experience symptoms ranging from febrile syndrome to neurological complications such as meningitis, meningoencephalitis, and encephalitis [7,9]. 

Since the discovery of USUV, only two human cases have been reported in Africa. These cases occurred in the Central African Republic in 1981 in a febrile patient with a rash and in Burkina Faso in 2004 in a 10-year-old patient with febrile jaundice [10]. The low number of human cases diagnosed in Africa makes it difficult to establish a clear clinical picture of the disease on the African continent. This arbovirus has gained the attention of the scientific community due to its recent incursions in Europe and due to the threat it represents for certain wild birds (mainly blackbirds and owls in Europe) [2]. Molecular and serologic evidence of USUV infection in European blood donors also suggests a silent spread of this virus among asymptomatic humans. To date, USUV infection has been reported in a dozen European countries, with more than a hundred cases of acute human infection being described, mainly in Europe [2,11]. Contrary to USUV, WNV has been described as responsible for many epidemics in Africa, Europe, and the United States [12]. On the African continent, cases of WNV have been reported in the Democratic Republic of Congo, Gabon, Namibia, and Tunisia [13]. The majority of symptomatic patients have fever, rash, headache, muscle and joint pain, or jaundice. Less than 1% of infections progress to neuroinvasive disease, with such progression being recorded in cases of immunocompetent or immunocompromised patients (meningitis, encephalitis, or acute flaccid paralysis) [13,14]. 

Clinical diagnosis is made difficult by the similarity of USUV and WNV symptoms with other flaviviruses or other infectious diseases. Diagnostic techniques used are RT-PCR, virus isolation in cell culture, and the detection of antiviral antibodies by Enzyme-linked immunosorbent assay (ELISA) tests. ELISA tests suffer from a lack of specificity due to cross-reactions that exist with flaviviruses [15]. To discriminate these viruses, positive ELISA samples must be systematically confirmed by microneutralization tests (MNT). There are no specific commercial tests for the differential diagnosis of USUV and WNV, making them difficult to monitor. Currently, in-house tests developed by reference laboratories are relied upon. The above-mentioned diagnostic problems make the monitoring of these viruses particularly difficult in resource-limited countries.

In Burkina Faso, the circulation of some flaviviruses such as dengue and yellow fever viruses (DENV and YFV) has been observed, notably with recurrent DENV epidemics since 2013 [16]. The country has recorded sporadic cases of yellow fever every year since the establishment of national surveillance in 2001. Since the identification of the first human case of USUV infection in Burkina Faso in 2004, no studies have been conducted to measure the extent of the circulation of this virus in the country. Hence the interest of our study, which aims to determine the seroprevalence of USUV and WNV in blood donations, animals (horses, dogs, chickens, and pigeons) and to perform molecular screening in patients with febrile fever and in *Culex quinquefasciatus* and *Aedes aegypti* mosquitoes the main vectors of arboviruses in Burkina Faso. 

## 2. Materials and Methods

### 2.1. Samples

#### 2.1.1. Blood Donors

The study involved blood donors from regional blood transfusion centers in the cities of Bobo-Dioulasso and Ouagadougou. Blood samples were collected from June to July 2020. A total of 501 samples were collected. This cohort harbors 114 (22.75%) females (median age: 28 years; interquartile range (IQR): 24–35.75 years) and 387 (77.25%) males (median age: 28 years; IQR 24–35 years). 

#### 2.1.2. Sera Specimens from Fever of Unknown Etiologies

188 samples from patients with fever of unknown etiology, including 23 with jaundice, were collected in 2019 and stored at −80 °C in the biobank of the MURAZ center in Bobo-Dioulasso to perform serological and molecular tests. 

#### 2.1.3. Sera Samples from Animals

Horses, dogs, chickens, and pigeons were collected from the towns of Bobo-Dioulasso and Ouagadougou. A total of 204 animal samples were collected from November 2021 to January 2022.

- A total of 81 horse sera samples (50 from Ouagadougou and 31 from Bobo-Dioulasso) were collected. The average age of the 81 horses analyzed was 7.11 years, with extremes ranging from 10 months to 27 years, corresponding to 79.01% of males and 20.99% of females. The samples were taken in the breeding sites by a veterinary technician. No horse was vaccinated against WNV, and all showed no symptoms at the time of sampling.
- A total of 52 dogs (25 from Ouagadougou and 27 from Bobo-Dioulasso) were included in our study. All samples were taken at the owners’ homes, and no dog showed symptoms. The average age of the dogs was 2.77 years, with extremes ranging from 2 months to 12 years. There were 34 males and 18 females.
- A total of 50 sera samples from chickens from traditional farms were collected in Ouagadougou (25) and Bobo-Dioulasso (25) and analyzed. The average age was 1.3 years, with extremes ranging from 5 months to 3 years. There were 10 males and 40 females. All samples were collected from poultry markets.
- A total of 21 domestic pigeons from traditional breeding were included in the study. We collected 10 sera samples in Ouagadougou and 11 in Bobo-Dioulasso. The average age of the pigeons was 12.8 months, with extremes ranging from 1 month to 2 years. There were 9 males and 12 females. All samples were collected from owners’ homes.

#### 2.1.4. Mosquito Samples

**Figure 1:**
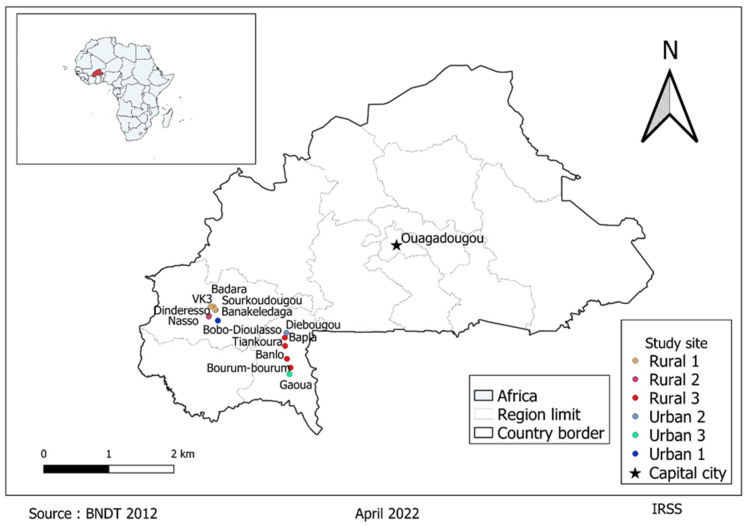
Location of mosquito collection sites in Burkina Faso. Each dot represents one sampling site; the samples from the sites that have the same dot color were grouped and renamed in rural 1 to 3 and in urban 1 to 3.

Mosquito collection was carried out in August and September 2019, June, July and October 2020, and May and June 2021. Mosquitoes were collected in the Hauts-Bassins and South-West regions of Burkina Faso (Figure 1). In the Hauts-Bassins region, the sampling localities were located on two road transects. The first transect was composed by the town of Bobo-Dioulasso, following four rural areas: Banakeledaga, Sourkoudougou, Badara and Vallée du Kou 3 (VK3). The sampling on the second transect were carried out in two rural forested areas: Nasso and Dinderesso. In the South-West region, the sampling was carried out in two urban areas (Diébougou and Gaoua) and in four rural sites (Bapla, Tiankoura, Banlo, and Bouroum-bouroum). Collection areas have been grouped and renamed in urban or rural areas.

Samples were carried out over two successive days in each locality. Three methods were used: the double net tent, the BG-Sentinel traps, and the prokopack aspiration. The various specimens (living) were identified morphologically using the identification keys [17,18,19], then killed cold and stored at −80 °C for subsequent analyses. 

#### 2.1.5. Mosquito Sorting and Crushing

No-blood-engorged females of *Culex quinquefasciatus* and *Aedes aegypti*, the main vectors of Arboviruses, were our species of interest for this study. As the sampling were composed of several genera and species of mosquitoes, a sorting was necessary to isolate the specimens of interest and to group them by area and by collection period. This sorting was carried out on an ice-cold platform to avoid viral genome degradation. Each species was placed in pools of 6 to 37 individuals per well in 96-well “deepwell” type. The crushing was carried out using the TissueLyser II (Qiagen (Hilden, Germany), 30 s at 30 Hz, 2 times). Beforehand, each pool was resuspended in 500 µL 1X-PBS (Phosphate Buffered Saline buffer (Thermo Fisher Scientific, Waltham, MA, USA) with two ice-cold steel bearing balls (3 mm diameter, LOUDET). After crushing, the supernatant was recovered for extraction of total RNA. 

### 2.2. Competitive Enzyme-Linked Immunosorbent Assay

The screening of human and animal sera was carried out with the ID Screen^®^ West Nile competition ELISA test, (Innovative diagnostic, Montpellier, France), which is a competition ELISA test. This test was originally developed to identify antibodies against the West Nile virus pr-E envelope protein, as it turned out to cross other flaviviruses and can therefore detect a large spectrum of flaviviruses including WNV and USUV and other related flaviviruses [20,21]. The test was performed according to manufacturer’s instructions.

### 2.3. Seroneutralization Assays

Viral microneutralization tests (MNT) were performed on cELISA positive sera to identify WNV or USUV infection for human, horse, and dog samples using USUV (France2018, MT863562) and WNV (lineage 2, MT863560) [21]. The sera were serially diluted in duplicate in 50 µL of Dulbecco’s Modified Eagle Medium (DMEM) of ThermoFisher supplemented by heat-inactivated fetal bovine serum 2% in a 96 plate, then with a dilution factor of 2 starting with 1/4th to 1/256th. USUV and WNV suspension at 200 tissue culture infectious dose 50 (TCID50) were then added in each well. After incubation for 90 min at 37 °C with 5% CO_2_, we added 100 µL of DMEM 2% containing 2000 vero cells per well. The plates were incubated at 37 °C with 5% CO_2_ for 5 days. Antibody titers were calculated by doing the reciprocal of the last dilution at which there is no cytopathic effects. Samples that were positive for both WNV and USUV were considered positive for either virus when the antibody titer for one virus is 4 times higher than the antibody titer for other virus.

### 2.4. USUV and WNV qRT-PCR

Nucleic acids were extracted from human sera using the automatic extractor MGISP-960, and amplification was performed with the LightCycler^®^ 480 from Roche according to the procedures described by Nikolay et al. for USUV [22] and Garcia et al. for WNV [23]. For mosquitoes, total RNA extraction was performed using the Biomek-FX machine (Beckman-Coulter) and the Nucleospin RNA virus extraction kit (Macherey-Nagel, Strasbourg, France), following the manufacturer’s instructions. A control of the quantity and quality of the RNA was measured by spectrophotometry (Nanodrop, Thermo Fisher Scientic, Waltham, MA, USA) and by capillary electrophoresis (Bioanalyser, Agilent Technologies, Santa Clara, CA, USA). The detection of USUV and WNV by real-time RT-qPCR was carried out using the LUNA Universal Probe One Step RT-qPCR Biolaps kit, respectively following the protocol described previously by Nikolay et al. and Tang et al. [22,24].

### 2.5. Statistical Analysis

In blood donors, the correlation between seroprevalence and independent variables such as origin, sex, and age were analyzed using a Pearson chi-square test or Fisher’s exact test. We also used the Odds ratio to show the association or lack thereof of independent variables with seroprevalence. Figures were made using GraphPad Prism 9 software and maps using QGIS 3.24.2 software.

### 2.6. Ethical Statement and Informed Consent

Study on human samples was approved by health research ethics committee of Burkina Faso (2020-3-049) and was performed in line with the regulations outlined in the Declaration of Helsinki. Data was anonymized for publication purposes. Animal sampling was performed according to national and institutional guidelines, informed written consent of animal owners was obtained, and sample collection was performed by veterinarians. 

Patients and blood donors were informed during the collection that their samples could be used later for other studies.

## 3. Results

### 3.1. WNV and USUV Infections in Humans Samples

In order to analyze the potential circulation of USUV and WNV in the general population in Burkina Faso, we decided to screen blood donor samples and clinical samples from the national YFV surveillance program. The seroprevalence of WNV and USUV was first evaluated in a collection of 501 samples from blood donors, using cELISA to detect prior flavivirus infection. A total of 389 samples were positive (77.65%, CI95%: 73.79–81.07) (Table 1 and Table S1). Samples positive for cELISA were then tested using MNT against WNV and USUV. Despite the inability to completely rule out antibody cross reactions, samples with close or equal neutralizing activity for WNV and USUV (<four-fold difference in MNT titer) were considered as potential co-infections. Among the positive samples, 71 were tested positive for USUV-specific antibodies (14.17%, CI95%: 11.39–17.49), and 96 for WNV-specific antibodies (19.16%, 95% CI: 15.95–22.83). A total of 52 samples demonstrated neutralizing activity for both USUV and WNV (Table 1). We did not find any significant associations between the origin, sex, and age of blood donors and USUV seroprevalence (Table 2A), whereas men had a higher seroprevalence for WNV than women (Table 2B). We additionally analyzed 188 fever samples of unidentified etiology for serological screening and using RT-qPCR for USUV and WNV. No WNV or USUV was detected in any of these samples.

### 3.2. WNV and USUV Infections in Domestic Animals

**Figure 2:**
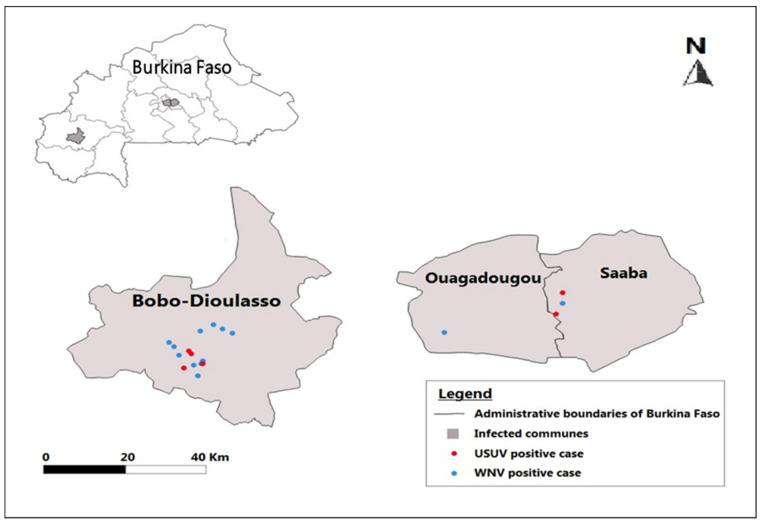
Geographical distribution of animals who tested positive for antibodies against USUV and WNV. Red dots represent USUV positive samples and blue dots WNV positive samples. In Bobo-Dioulasso, all the positives are concentrated in the same area.

Arboviruses are maintained in sylvatic and urban cycles through non pathological infection in natural reservoirs but can also affect other animal hosts. To monitor the potential infection in domestic animals, we screened various species for the presence of specific USUV and WNV antibodies. Animal samples were collected in different neighborhoods of the two major cities of Burkina Faso (Ouagadougou and Bobo-Dioulasso). All animals were from domestic breeding. A total of 81 horses, 52 dogs, 50 chickens and 21 pigeons were included in our study. Among the 81 horses analyzed, we obtained 73 horses positive for cELISA (48 from Ouagadougou and 25 from Bobo-Dioulasso). Among them, 5 (6.17%, CI95%: 2.66–13.64) were positive for USUV antibodies, and 14 (17.28%, CI95%: 10.58–26.94) for WNV (Table 3). We identified anti-flavivirus antibodies in 14 dogs (10 from Ouagadougou and 4 from Bobo-Dioulasso); only one of the dogs presented neutralizing activity for WNV; and all dog samples were negative for USUV antibodies. Concerning avian analyses, we identified anti-flavivirus antibodies in 4 chicken samples but no positives for USUV and WNV after MNT tests. In pigeons, of the two samples that gave a positive result for the cELISA test, we detected one sample presenting a USUV positive antibody and another one presenting anti-WNV antibodies (Table 3). The majority of positive animals came from Bobo-Dioulasso (Table 4, Figure 2).

### 3.3. WNV and USUV Screening in Mosquitoes

A total of 1356 Aedes aegypti and 1659 Culex quinquefasciatus no-blood-engorged females were obtained after sorting. Blood-fed mosquitoes can contain viruses in the blood that are not mosquito borne and thus lead to false conclusions on the viral pathogens borne by mosquitoes in the study area. 

These species were most dominant in the city of Bobo-Dioulasso (Urban 1) (Figure S1). We did not identify the presence of USUV or WNV in these samples. To validate the negative results of our RT-qPCR, we always added a positive sample (RNA isolated from cell culture infections) in our PCR plates. Moreover, we have analyzed in parallel and with the same RT-qPCR a batch of mosquitoes from France [25]. Our results with the French samples show that our RT-qPCR is able to detect both viruses and allows to identify positive samples for virus isolation and genome sequencing. Finally, the Burkinabe samples were also screened with a RT-qPCR targeting the mosquito 5.8S ribosomal RNA to validate the absence of PCR inhibitors.

## 4. Discussion

There was no previous data regarding the circulation of USUV and WNV in Burkina Faso. The only human case of West African USUV infection was identified in Burkina Faso in 2004, which suggests that this virus circulated in the country several years ago [10]. Since 2014, Burkina Faso has developed a national reference laboratory for hemorrhagic fevers, which is responsible for monitoring arbovirus infections in humans. This monitoring is mainly concerned with DENV, YFV, zika and chikungunya viruses, with no analysis of USUV or WNV having been performed thus far. Notably, a modeling study has identified a number of countries (Togo and Benin) bordering Burkina Faso as areas at risk of the emergence of WNV [12]. 

To have a general view of the potential circulation of these viruses, our study targeted several epidemiologically relevant hosts: humans, horses, dogs, birds, and mosquitoes, and highlighted the active circulation of USUV and WNV in Burkina Faso. We found the presence of antibodies directed against these two viruses in blood donors, horses, dogs and pigeons. Several other studies have shown the co-circulation of USUV and WNV viruses in humans and animals, particularly in Europe [26,27]. Horses, dogs, domestic birds and chickens have been identified as accidental hosts of USUV and WNV [7,20,28,29,30,31]. Horses are known as a species particularly susceptible to WNV and USUV infection, and are considered as valuable sentinels of the risk of WNV epizootic transmission [32,33]. In this species, we observed a high seroprevalence of WNV (17.28%) compared to USUV (6.17%). In general, WNV is known to be more widespread than USUV [26], and our study appears to confirm the same trend in Burkina Faso as well. That said, some studies have shown a higher circulation of USUV in humans in regions where the two viruses are endemic [21]. During the collection of our samples, horse breeders reported the occurrence in some horses of neurological syndromes typically associated with infection. Unfortunately, this information was not documented, nor does such data exist at the level of veterinary clinics in the country. It would be very informative to set up a surveillance system for flaviviruses in horses, with the collaboration of veterinary clinics and horse owners in Burkina Faso. Some previous studies have reported high seroprevalence of WNV in horses in sub-Saharan Africa, particularly in Ivory Coast, Nigeria and Chad [34,35]. Another study carried out in Morocco reported serological evidence of the circulation of USUV and WNV in horses but also in dogs [29]. Although dogs are not described as developing clinical signs after USUV and WNV infection, our study included them as relevant candidates for surveillance due their proximity to humans and their high exposure rates to WNV, as has been previously documented [36]. While our study we observed only one dog presented antibodies against WNV and none had antibodies to USUV, it must be acknowledged that our cohort was small in number to be conclusive. The small sample size of pigeons (21) collected did not allow us to measure the real extent of the circulation of these viruses in this population of domestic birds although it did allow us to detect positive birds for USUV and WNV. The regular displacement of this species suggests that they could be more exposed than mammals to infections by these viruses. To date, there have been very few studies on domestic pigeons, even though the first WNV infection in birds was detected in organs and blood from a pigeon in 1955 [13]. A WNV surveillance system based on the serological testing of domestic pigeons in Greece reported seroprevalences of 54% in 2010 and 31% in 2011, with one pigeon also testing positive for anti-USUV antibodies during the same period [37]. Also, an epidemiological survey on zoonoses in Spain identified the circulation of USUV and WNV in feral pigeons [38]. Our study is the first to show serological evidence of USUV in domestic pigeons in West Africa. A WNV serological survey of domestic pigeons in Nigeria reported a seroprevalence of 3.5% [39]; which is close to the 4.76% rate found in our study. Another study conducted in Mali showed the presence of USUV and WNV antibodies in other species of domestic bird (duck and chicken) [30]. A large-scale study on domestic pigeons in Burkina Faso would make it possible to better situate their share in the circulation of USUV and WNV. It should be noted that samples from domestic pigeons are more easily accessible compared to samples from wild birds. In our study, all chickens were found to be negative for antibodies to both viruses. Previous studies estimate that USUV has a limited pathogenicity in domestic chickens [40], while WNV antibodies have been identified in chickens by authors in Turkey and Croatia [31,41].

USUV and WNV have been isolated from several mosquito species of the genera *Culex* and *Aedes* [4], and some *Culex* and *Aedes* species have been shown as capable of transmitting USUV and WNV in Africa [13,42]. The presence of these vectors has already been demonstrated in Burkina Faso [43]. In the course of our study, we searched for USUV and WNV in two mosquito species using RT-PCR: *Culex quinquefasciatus* (1659 specimens) and *Aedes aegypti* (1356 specimens). The PCR was negative for the viruses sought in these two species of mosquitoes, but also for other viruses such as DENV, zika, and chikungunya viruses known to be circulating in Burkina Faso. [44]. The data for the absence of detection of other flaviviruses (DENV and ZIKV) in mosquitoes have been recently published and are thus available [45]. Moreover, virus positive mosquitoes are extremely scarce for most epidemiological situations involving arboviruses. These results show a low prevalence of the circulation of USUV and WNV in vector population, hence the difficulty of identifying and monitoring these viruses in mosquitoes. A more exhaustive mosquitoes sampling or targeted mosquito collection in localities where we identified animals positive to both viruses would increase the likelihood to find the positive specimens in the vector population.

There is currently an information gap as to the circulation of USUV and WNV in humans in West Africa. For example, concerning WNV, there are more than twenty African countries (including Burkina Faso) in which no seroprevalence study has yet been conducted in humans to measure the extent of the circulation of the virus [46]. Two studies in Africa have considered the seroprevalence of WNV in blood donors. In Egypt, a study reported a high seroprevalence of 55%, and in Rwanda, a seroprevalence of 10.4% [47,48]. No data exists on the seroprevalence of USUV among blood donors in Africa. The only available data concerning blood donors derives from studies carried out in Europe. Of those studies, several have reported quite low seroprevalences of USUV among blood donors in Italy (1% and 0.23%), Germany (<1%), and Hungary (<1%) [49,50,51,52]. These results are all very far from the 14.17% obtained in our study, suggesting a significant level of circulation of USUV virus in humans in Burkina Faso. We observed a statistically significant association between blood donor gender and WNV seroprevalence as men had a high seroprevalence compared to women. One hypothesis to explain this is that in Burkina Faso, due to their typical daily activities, men spend more time outside the home than women, which would expose them more to mosquito bites. The search for viral RNA of USUV and WNV using RT-PCR in fever specimens of unknown etiologies returned negative results for all samples analyzed (188). In Burkina Faso, people often resort to self-medication when they are sick, and all febrile illnesses are generally treated as malaria. Therefore, people only consult a doctor when self-medication does not work. This late consultation makes it difficult to detect the virus in the blood because during USUV or WNV infection, the viremia window is very short in humans [2]. It is therefore necessary to take a blood sample very early during disease onset to increase the probability of detecting the virus in the blood.

In our study, 52 human samples demonstrated neutralizing activity for both USUV and WNV. These results do not allow us to conclude whether they are simultaneous infections or infections acquired at different times. Co-infections have already been documented in other studies in birds and mosquitoes [3,53]. The ability of vectors to transmit multiple arboviruses in a single bite increases the likelihood of individuals being infected with multiple arboviruses simultaneously [54]. Such co-infections would further complicate the interpretation of serological tests and could affect the performance of molecular tests.

## 5. Conclusions

To conclude, our study shows an active spread of USUV and WNV in Burkina Faso in humans and horses, especially for WNV. This study highlights the value of developing monitoring programs to better prevent, detect, and warn about USUV and WNV circulation, in both primary and incidental hosts. Developing surveillance programs in Burkina Faso would help to anticipate the veterinary and public health risks associated with these viruses and further studies would be needed to characterize these viruses in wild birds to determine their potential origin.

## Figures and Tables

**Table 1:** cELISA test and MNT results in blood donors among the 501 human samples analyzed.

Variable	cELISA Positive n (%)	MNT Positive n (%)		
		USUV+	WNV+	USUV + WNV+
Total	389 (77.64)	71 * (14.17)	96 * (19.16)	52 * (10.38)
Origin				
Ouagadougou	216 (84.37)	44 (17.18)	50 (19.53)	31 (12.10)
Bobo-Dioulasso	173 (70.61)	27 (11.02)	46 (18.77)	21 (8.57)
Gender				
Male	302 (78.03)	56 (14.47)	82 (21.18)	45 (11.62)
Female	87 (76.31)	15 (13.15)	14 (12.28)	7 (6.14)
Age				
18–24	130 (76.47)	24 (14.11)	33 (19.41)	17 (10.0)
25–34	146 (74.11)	24 (12.18)	30 (15.22)	15 (7.65)
35–44	81 (85.26)	17 (17.89)	23 (24.21)	14 (14.73)
45–59	32 (82.05)	6 (15.38)	10 (25.64)	6 (15.38)

* among the 71 USUV positives and 96 WNV positives, there are 52 samples positive for both USUV and WNV.

**Table 2:** (**A**). USUV seroprevalence according to the socio-demographic characteristics of the blood donors. (**B**). WNV seroprevalence according to the socio-demographic characteristics of the blood donors.

**(A)**			
**Variable**	**Positive n (%)**	**Odds Ratio IC95%**	***p*-Value**
Origin			0.0547
Ouagadougou	44 (17.18)	1	
Bobo-Dioulasso	27 (11.02)	0.6 [0.36–1]	
Gender			0.8786
Male	56 (14.47)	1	
Female	15 (13.15)	0.9 [0.49–1.66]	
Age			0.6213
18–24	24 (14.11)	1	
25–34	24 (12.18)	0.84 [0.46–1.54]	
35–44	17 (17.89)	1.33 [0.67–2.62]	
45–59	6 (15.38)	1.11 [0.42–2.93]	
**(B)**			
**Variable**	**Positive n (%)**	**Odds Ratio IC95%**	***p*-Value**
Origin			0.9097
Ouagadougou	50 (19.53)	1	
Bobo-Dioulasso	46 (18.77)	0.95 [0.61–1.49]	
Gender			0.0415 *
Male	82 (20.15)	1	
Female	14 (12.28)	0.52 [0.28–0.96]	
Age			0.2040
18–24	31 (18.23)	1	
25–34	29 (14.72)	0.75 [0.44–1.29]	
35–44	22 (23.15)	1.33 [0.73–2.45]	
45–59	10 (25.64)	1.43 [0.63–3.22]	

* *p* < 0.05.

**Table 3:** USUV and WNV MNT results in cELISA positive animals.

Species	Number of Specimens	cELISA	MNT Antibodies Titer		Conclusion
			USUV	WNV	
Horses	81	73	128	128	USUV + WNV+
			32	Negative	USUV+
			32	256	WNV+
			64	64	USUV + WNV+
			8	Negative	USUV+
			8	Negative	USUV+
			32	128	WNV+
			Negative	128	WNV+
			Negative	128	WNV+
			Negative	32	WNV+
			Negative	32	WNV+
			Negative	8	WNV+
			Negative	256	WNV+
			Negative	16	WNV+
			Negative	32	WNV+
			Negative	16	WNV+
			Negative	16	WNV+
Dogs	52	14	0	64	WNV+
Chickens	50	4	Negative	Negative	Negative
Pigeons	21	2	8	Negative	USUV+
			0	8	WNV+

**Table 4:** Seroprevalence of USUV and WNV according to animal location.

Species	Results	Total	Origin	
			Ouagadougou	Bobo-Dioulasso
Horses	USUV + n (%)	5 (6.17%)	2 (4%)	3 (9.67%)
	WNV + n (%)	14 (17.28%)	1 (2%)	13 (41.93)
Dogs	USUV + n (%)	0 (00%)	0 (00%)	0 (00%)
	WNV + n (%)	1 (1.92%)	1 (4%)	0 (00%)
Chickens	USUV + n (%)	0 (00%)	0 (00%)	0 (00%)
	WNV + n (%)	0 (00%)	0 (00%)	0 (00%)
Pigeons	USUV + n (%)	1 (4.76%)	0 (00%)	1 (9.09%)
	WNV + n (%)	1 (4.76%)	0 (00%)	1 (9.09%)

## Data Availability

Not applicable.

## Supplementary Materials

The following supporting information can be downloaded at: https://www.mdpi.com/article/10.3390/microorganisms10102016/s1, Figure S1: Number of mosquito after sorting; Table S1: Socio-demographic characteristics of bloods donors samples.
