# Supplementary material for: Screening of Circulation of Usutu and West Nile Viruses: A One Health Approach in Humans, Domestic Animals and Mosquitoes in Burkina Faso, West Africa
Source: Microorganisms. 2022 Oct 12;10(10):2016. doi: 10.3390/microorganisms10102016 (PMC9610586; doi:10.3390/microorganisms10102016)
Supplement: Supplementary file 1 [file microorganisms-10-02016-s001.zip › microorganisms-1955383-supplementary.pdf]

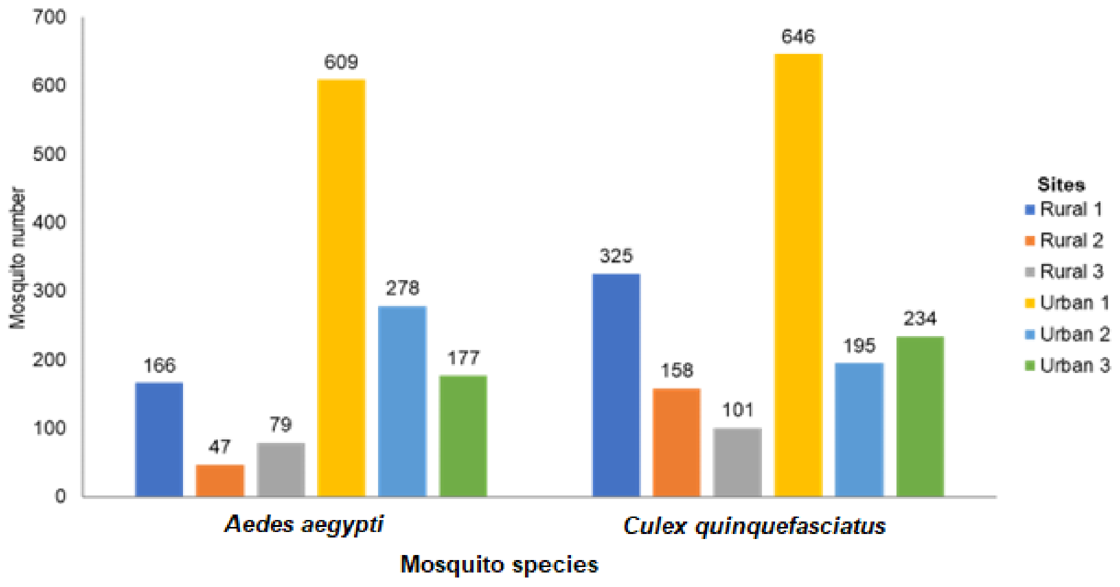

Figure S1: Number of mosquitoes after sorting.

Table S1: Socio-demographic characteristics of blood donor samples.

**Variable**	**n(%) or median**		
	**Ouagadougou**	**Bobo-Dioulasso**	**Total**
	**256**	**245**	**501**
**Gender**			
Male	183 (71.48%)	204 (83.27%)	387 (77.25%)
Female	73 (28.52%)	41 (16.73%)	114 (22.75%)
**Age**			
18-24	72 (28.12%)	98 (40%)	170 (33.94%)
25-34	94 (36.72%)	103 (42.04%)	197 (39.32%)
35-44	65 (25.39%)	30 (12.25%)	95 (18.96%)
45-59	25 (9.77%)	14 (5.71%)	39 (7.78%)
